# Supplementary material for: Prospects for Prostate Cancer Chemotherapy: Cytotoxic Evaluation and Mechanistic Insights of Quinolinequinones with ADME/PK Profile
Source: Biomedicines. 2024 Jun 3;12(6):1241. doi: 10.3390/biomedicines12061241 (PMC11200585; doi:10.3390/biomedicines12061241)
Supplement: Supplementary file 1 [file biomedicines-12-01241-s001.zip › biomedicines-2931278-SI.pdf]

Prospects for Prostate Cancer Chemotherapy: Cytotoxic Evaluation and Mechanistic Insights of Quinolinequinones with ADME/PK Profile

Ayse Tarbin Jannuzzi [1], Ayse Mine Yilmaz Goler [2], Abanish Biswas [3], Subodh Mondal [4], Vinay N. Basavanakatti [5], Hatice Yıldırım [6], Mahmut Yıldız [7], Nilüfer Bayrak [8], Venkatesan Jayaprakash [3] and Amaç Fatih TuYuN [8,*]

[1] Department of Pharmaceutical Toxicology, Faculty of Pharmacy, İstanbul University, 34116 İstanbul, Türkiye; tarbin.cevik@istanbul.edu.tr
[2] Department of Biochemistry, School of Medicine/Genetic and Metabolic Diseases Research and Investigation Center, Marmara University, 34854 İstanbul, Türkiye; aysemine.yilmaz@gmail.com
[3] Department of Pharmaceutical Sciences & Technology, Birla Institute of Technology, Mesra, Ranchi 835215, Jharkhand, India; abanish37@gmail.com (A.B.); drvenkatesanj@gmail.com (V.J.)
[4] Bioanalysis, Eurofins Advinus BioPharma Services India Pvt Ltd., Bengaluru 560058, Karnataka, India; subodh.mondal@advinus.com
[5] Adgyl Lifesciences Private Limited, Bengaluru 560058, Karnataka, India; vinay.b@advinus.com
[6] Department of Chemistry, Engineering Faculty, Istanbul University-Cerrahpasa, Avcılar, 34320 İstanbul, Türkiye; hyildirim@iuc.edu.tr
[7] Department of Chemistry, Gebze Technical University, Gebze, 41400 Kocaeli, Türkiye; yildizm@gtu.edu.tr
[8] Department of Chemistry, Faculty of Science, Istanbul University, Fatih, 34134 İstanbul, Türkiye; nbayrak@istanbul.edu.tr
* Correspondence: aftuyun@gmail.com or aftuyun@istanbul.edu.tr; Tel.: +90-212-440-0000

Contents

ADME and PK Profiling 3-7

Experimental Section

S2. ADME and PK Profiling

S2.1. In vitro Metabolic Stability Study

Liver microsomes of mouse (Cat. No. M1000, Lot. No. 1710069), rat (Sprague-Dawley, male, Cat. No. R1000, Lot. No. 1610290), dog (Beagle, male, Cat. No. D1000, Lot. No. 1310086) and human (Cat. No. H0610, Lot. No. 1610016) of Xeno Tech LLC, Kansas, USA were used for testing the metabolic stability of **AQQ1** and **AQQ2**. A final protein (liver microsomal) concentration of 0.5 mg/mL and final test substance (**AQQ1** and **AQQ2**) concentration of 0.5 μM were used for the study. To the respective microsomes at a volume of 12.5 μL in the well, 2.5 μL of test substance (200 uM in acetonitrile: dimethyl sulfoxide, 96:4) in the presence and absence of NADPH (50 μL, 10 mM) were added and the final volume made up to 500 μL with sodium phosphate buffer (50 mM, pH 7.4). The one without NADPH served as control. Sampling (50 μL) were done at the following time points: 0, 5, 10, 15, and 30 min for sample and at 0 and 30 min for control. Acetonitrile (150 μL) was used as quenching solvent, internal standard (Rolipram) was added, vortexed/centrifuged and the aliquot from the supernatant was analyzed using LC-MS/MS. All the experiments were performed in duplicate.

S2.2. In vivo Bioavailability Study of AQQ1 and AQQ2 in Male Sprague-Dawley Rats

Sprague-Dawley rats (HyLasco Biotechnology (India) Pvt. Ltd., a subsidiary of Charles River from the US) of 8-12 weeks of age (at the time of treatment), weighing between 220 and 320 g, were used in this study.

Housing: Animals were housed in polysulfonate cages in a typical research laboratory environment at 25 ± 3°C and 50%-70% relative humidity with approximately 12 h light and dark cycles maintained with an enrichment device. During the study, the animals had access to a rat maintenance diet (Altromin Spezialfutter GmbH, Germany) and purified water (UV-treated, charcoal- filtered).

Animal experiments were conducted in accordance with the guidelines of the Committee for the Purpose of Control and Supervision of Experiments on Animals (CPCSEA), Ministry of Social Justice and Environment, Government of India, and approved by the Institutional Animal Ethics Committee. All rats were cannulated with the jugular vein, fasted overnight before dosing (except IV dose), and had access to food and water for ~4 h post-dosing. **AQQ1** and **AQQ2** in NMP (10%) + PBS (pH 7.4) qs was administered intravenously at a dose of 1 mg/kg bw (10 mL/kg). **AQQ1** and **AQQ2** in NMP (10%) + Cremophor EL (5%) + PEG400 (30%) + PG (20%) + PBS (pH 7.4) qs was administered PO at a dose of 5 mg/kg bw (10 mL/kg). The animal weight on the day of dosing was used to calculate the required volume of the formulation and administered to the rats. After dosing, blood samples (~0.250 mL) were collected from the jugular vein at the following time points: 0.083 (IV only), 0.25, 0.5, 1, 2, 4, 6, 8, and 24 h using a serial sampling design (equal volumes of heparinized saline were replaced after each sample collection). The samples were collected in pre-labeled microcentrifuge tubes containing K_2EDTA (20 µL of 200 mM solution

per mL of blood) as an anticoagulant. The blood samples were stored at -60°C until bioanalysis using the fit-for-purpose LC-MS/MS method. Pharmacokinetic parameters were calculated using the non-compartmental analysis (NCA) tool of the validated Phoenix® WinNonlin® 8.3.

**Treatment Group**	**Treatment/Route**	**Dose (mg/kg)**	**Dose volume (mL/kg)**	**Formulation Stregth (mg/mL)**
G1	**AQQ1** (IV)	1 mg/kg	10	0.1
G2	**AQQ2** (IV)	1 mg/kg	10	0.1
G3	**AQQ1** (PO)	5 mg/kg	10	0.5
G4	**AQQ2** (PO)	5 mg/kg	10	0.5

S2.3. Bioanalytical Method (LC/MS/MS) for AQQ1 **and** *AQQ2*

S2.3.1. Chemicals and Reagents

MS-grade (99.0% pure) ammonium formate and formic acid were obtained from Sigma Aldrich. HPLC-grade acetonitrile (ACN) and dimethyl sulfoxide solvents were purchased from Merck, Germany. Milli-Q® water used for the preparation of the mobile phase, rinsing solvent, and seal washes was obtained from the in-house (Eurofins Advinus Limited) Milli-Q® system. Cremophor EL, PEG 400, and PBS were obtained from Sigma Aldrich, Bengaluru, Karnataka, India. Similarly, Propylene Glycol was procured from Fischer Scientific and NMP from Spectrochem, Bengaluru, Karnataka, India. The internal standard MQD24 ($C_{17}H_{13}ClN_2O_3$, 328.74, purity >97%) used in this study was procured from Sigma-Aldrich. A SCIEX API 4000™ LC/MS/MS triple quadrupole mass spectrometer system equipped with a negative electrospray ionization (ESI) source and Shimadzu prominence HPLC comprising binary pumps, a column oven, and an SIL-HTC autosampler was used in this study. Data acquisition, integration, and quantification were performed using Analyst® 1.6.3.

Chromatographic and mass spectrometric conditions Liquid chromatographic separations of **AQQ1**, **AQQ2**, and the internal standard, MQD24, were achieved on a reverse-phase Synergi Fusion RP 50 × 4.6 mm, 5 μm column operating at 40 °C. The gradient mobile phase was used starting with an initial 90:10, 3 min:10:90, 6 min:10:90, and 6–10 min:90:10 for up to 8 min. Additionally, 5 mM ammonium formate with 0.1% formic acid (Mobile phase and 0.1% formic acid in Acetonitrile (Mobile phase was delivered at a flow rate of 0.6 mL/min without a splitter.

The mass spectrometer was operated in negative electrospray ionization mode with unit mass resolution in a quadrupole analyzer with a dwell time of 200 ms, and the analytes were detected

using multiple reaction monitoring (MRM). The compound parameters for **AQQ1**, **AQQ2**, and MQD24 (internal standard) were optimized along with the MRM transition (m/z) to achieve sensitivity. The source parameters were optimized to a curtain gas N_2 flow of 25 psi (CUR), nebulizer N_2 gas at 40 psi (gas 1), ion spray voltage of +5500 V (IS), auxiliary N_2 gas of 60 psi (gas 2) with a turbo spray temperature of 450°C (TEMP), and collision-activated dissociation gas (CAD) of 10 psi. The MRM transition (m/z) selected for the analyte **AQQ1** was 330.20 and 299.30, for the analyte **AQQ2** was 343.20 and 298.30, and for the analyte MQD24 (internal standard) was 367.10 and 332.20. A system suitability test was performed prior to sample analysis. The system suitability test comprised six replicate injections of the extracted ULOQ and an extracted blank and LLOQ sample from rat plasma. The percentage coefficient of variation (CV (%)) for the peak area ratio (analyte to internal standard) of six replicate injections was <5%, meeting the acceptance criteria. The retention time was within ±0.5 min variation in each analytical run.

Sample preparation: **AQQ1**, **AQQ2**, and MQD24 (internal standard) were extracted from rat plasma samples using the protein precipitation (crashing) method. To 50 µL of CC/ QC/study samples, 200 µL of Internal Standard Working Solution in acetonitrile was added to all the tubes except for the Standard Blank sample and vortexed to mix. Vortex (Vibramax 100, Heidolph Instruments) was applied for approximately 10 min. The samples were centrifuged (Eppendorf 5810R) for 10 min at 10,000 rpm at set temperature of 4°C. A sufficient volume of supernatant was aliquoted into autosampler vials for LC–MS/MS analysis (SCIEX API 4000).

S2.3.2. Preparation of Calibration Standards And Quality Control Samples

Stock solutions of **AQQ1**, **AQQ2**, and MQD24 (internal standards) were prepared in dimethyl sulfoxide (DMSO) and acetonitrile at a concentration of 1 mg/mL. The stock solution of **AQQ1**

and **AQQ2** were further diluted using DMSO to prepare calibration standard solutions in the concentration range of 23-25,800 ng/mL. Acetonitrile was used as an internal standard to prepare a working solution with a concentration of 500 ng/mL. These solutions were then spiked into interference-free rat blank plasma to obtain calibration standards in the pharmacologically relevant range (1.15-1290 ng/mL). Similarly, the quality control (QC) samples were prepared using independent stock solutions of analytes to obtain concentrations of 3.00, 500, and 1000 ng/mL in rat plasma, representing low, medium, and high concentration QC samples, respectively. Stock solutions diluted standard solutions, quality control solutions, and internal standard solutions were stored at 2-8 °C. Spiked plasma samples (calibration standards and quality controls) were freshly prepared prior to sample analysis.
